# To Remove Fish-Bones from the Throat

**Published:** 1883-09

**Authors:** 


### To Remove Fish-Bones From the Throat.

To remove fish-bones from the throat, Prof. Voltolini, at Breslau, recommends a gargle composed of muriatic acid, 4 parts; and water, 240 parts. The teeth have to be protected by lard or oil. The fish-bones become flexible, and they disappear entirely after a short time.

Is it a Fact? At the meeting of the Western Academy at Madison, Wisconsin, Dr. R. Ludlam, of Chicago, stated as a clinical fact, that he had never seen a patient who had leucorrhœa during pregnancy troubled with morning sickness. Let us hear from some of our specialists.

We learn that the authorities of the Madras Medical College have lately established a chair of Dental Surgery, and have appointed to the professorship Mr. H. J. Gould, L.D.S. Eng., formerly a student at the Dental Hospital of London.

